# Stellettin B Sensitizes Glioblastoma to DNA‐Damaging Treatments by Suppressing PI3K‐Mediated Homologous Recombination Repair

**DOI:** 10.1002/advs.202205529

**Published:** 2022-12-01

**Authors:** Xin Peng, Shaolu Zhang, Yingying Wang, Zhicheng Zhou, Zixiang Yu, Zhenxing Zhong, Liang Zhang, Zhe‐Sheng Chen, Francois X. Claret, Moshe Elkabets, Feng Wang, Fan Sun, Ran Wang, Han Liang, Hou‐Wen Lin, Dexin Kong

**Affiliations:** ^1^ Tianjin Key Laboratory of Technologies Enabling Development of Clinical Therapeutics and Diagnostics School of Pharmacy Tianjin Medical University Tianjin 300070 China; ^2^ Key Laboratory of Immune Microenvironment and Diseases (Ministry of Education) Tianjin Medical University Tianjin 300070 China; ^3^ Department of Bioinformatics and Computational Biology The University of Texas MD Anderson Cancer Center Houston TX 77030 USA; ^4^ Department of Systems Biology the University of Texas MD Anderson Cancer Center Houston TX 77030 USA; ^5^ Department of Pharmacology and Chemical Biology State Key Laboratory of Oncogenes and Related Genes Shanghai Jiao Tong University School of Medicine Shanghai 200025 China; ^6^ Department of Pharmaceutical Sciences College of Pharmacy and Health Sciences St. John's University Queens NY 11439 USA; ^7^ The Shraga Segal Department of Microbiology Immunology and Genetics Faculty of Health Sciences Ben‐Gurion University of the Negev Beer‐Sheva 84105 Israel; ^8^ Department of Genetics School of Basic Medical Sciences Tianjin Medical University Tianjin 300070 China; ^9^ Research Center for Marine Drugs State Key Laboratory of Oncogenes and Related Genes Department of Pharmacy Renji Hospital School of Medicine Shanghai Jiaotong University Shanghai 200127 China

**Keywords:** DNA‐damaging treatment, glioblastoma, homologous recombination repair, PI3K, stellettin B

## Abstract

Glioblastoma (GBM) is the most aggressive type of cancer. Its current first‐line postsurgery regimens are radiotherapy and temozolomide (TMZ) chemotherapy, both of which are DNA damage‐inducing therapies but show very limited efficacy and a high risk of resistance. There is an urgent need to develop novel agents to sensitize GBM to DNA‐damaging treatments. Here it is found that the triterpene compound stellettin B (STELB) greatly enhances the sensitivity of GBM to ionizing radiation and TMZ in vitro and in vivo. Mechanistically, STELB inhibits the expression of homologous recombination repair (HR) factors BRCA1/2 and RAD51 by promoting the degradation of PI3K*α* through the ubiquitin‐proteasome pathway; and the induced HR deficiency then leads to augmented DNA damage and cell death. It is further demonstrated that STELB has the potential to rapidly penetrate the blood‐brain barrier to exert anti‐GBM effects in the brain, based on zebrafish and nude mouse orthotopic xenograft tumor models. The study provides strong evidence that STELB represents a promising drug candidate to improve GBM therapy in combination with DNA‐damaging treatments.

## Graphical abstract

Glioblastoma is one of the most lethal types of cancer. The current first‐line post‐surgery regimens are radiotherapy and temozolomide chemotherapy, both of which are DNA damage‐inducing therapies. The study provides strong evidence that stellettin B, a triterpene compound isolated from marine sponges *Jaspis stellifera*, represents a promising drug candidate to improve Glioblastoma therapy in combination with the two DNA‐damaging treatments.

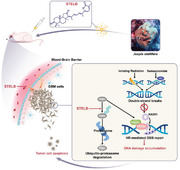

## 1 Introduction

Glioblastoma (GBM) is the most common intracranial malignancy that accounts for more than half of all primary gliomas and is also the most lethal cancer type.^[^ ^1^ ^]^ Even with maximal safe surgical resection, radiotherapy, and chemotherapy, the median progression‐free survival and overall survival time after initial diagnosis are only 6 and 15 months, respectively.^[^ ^2^ ^]^ Because of this dismal prognosis, tremendous efforts have been made to improve the clinical outcome of patients with GBM. Unfortunately, over the past decade, all strategies with promising results in preclinical studies have failed to demonstrate an overall survival benefit in large randomized clinical trials.^[^ ^3^ ^]^

GBM is highly heterogeneous at the cellular and molecular levels, and thus, satisfactory anti‐tumor effects are hard to achieve using small molecule drugs or monoclonal antibodies that only have a limited number of targets.^[^ ^4^ ^]^ The current post‐surgery regimens of GBM still largely rely on broad‐spectrum DNA‐damaging treatments, such as conventional radiotherapy based on ionizing radiation (IR) and temozolomide (TMZ) chemotherapy.^[^ ^5^ ^]^ The lack of response to these two standard treatments is mostly due to inherent or acquired resistance of GBM to DNA lesions.^[^ ^6^ ^]^ Therefore, there is an urgent clinical need to develop new drugs to overcome the resistance of GBM to DNA damage‐inducing therapies.

Natural products are a highly valuable source for the development of novel therapeutics because of their accumulated evolutionary changes and structural novelty and diversity.^[^ ^7^ ^]^ The isomalabaricane triterpenoid family of chemicals, a rare class of marine natural products, has attracted wide attention due to their remarkable and specific anticancer properties.^[^ ^8^ ^]^ However, their natural scarcity and unknown mode of action have impeded their development into clinical drugs. Recently the Sarlah group has successfully synthesized stellettin E (STELE) and rhabdastrellic acid A,^[^ ^8^ ^]^ removing a major barrier in drug development for the whole family of chemicals. Notably, previous structure‐activity relationship studies have shown that stellettin A (STELA), stellettin B (STELB), and STELE share a singular *trans‐syn‐trans*perhydrobenz[e]indene core that is indispensable for their anticancer effects.^[^ ^9^ ^]^ Therefore, the stellettin compounds represent attractive anticancer drug candidates for in‐depth investigation.

We previously isolated STELB from an extract of the marine sponge *Jaspis stellifera* and examined its antitumor effect using a JFCR39 cancer cell line panel.^[^ ^10^ ^]^ STELB shows highly potent inhibitory activities against a subset of cancer cells.^[^ ^10^, ^11^ ^]^ In particular, for GBM cell lines such as SF295, it exhibits ≈1000‐fold selectivity over normal cells,^[^ ^10a^ ^]^ suggesting mechanistic specificity rather than unbridled toxicity. Inspired by these preliminary results, here we aimed to investigate the anti‐GBM effects of STELB as a sensitizing agent to DNA‐damaging treatments, including IR and TMZ, and elucidate the underlying mechanisms.

## 2 Results

### 2.1 STELB Increases the Sensitivity of GBM Cells to IR

**Figure 1:**
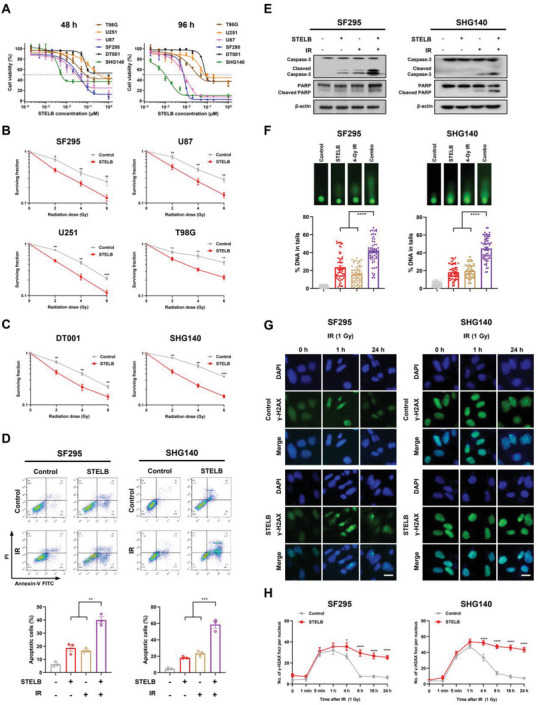
STELB sensitizes GBM cells to IR. A) Cell viability assays for established GBM cell lines and primary patient‐derived GBM cell lines treated with increasing concentrations of STELB for 48 h or 96 h. B,C) Clonogenic survival curves for GBM cell lines pretreated with STELB (0.03 × 10^−6^ m for SHG140, SF295 and U87, 0.1 × 10^−6^ m for U251 and T98G, 0.2 × 10^−6^ m for DT001; the same concentrations were used in subsequent cellular experiments in all main figures and supplementary figures unless indicated) for 24 h followed by exposure to IR at doses from 0 to 6 Gy. D) Apoptosis assays of SF295 and SHG140 cells pretreated with STELB for 24 h and then irradiated at 4 Gy. Apoptosis was detected by Annexin V/PI staining after 72 h. FACS quantification of the total apoptotic cell population, including Annexin V^+^/PI^−^ early apoptotic cells and Annexin V^+^/PI^+^ late apoptotic cells. E) Western blot analysis of Caspase‐3 and PARP in SF295 and SHG140 cells after treatment with STELB and/or IR for 48 h. F) Alkaline comet assays of SF295 and SHG140 cells treated with STELB and/or IR for 48 h to measure both single‐ and double‐strand DNA breaks. The % DNA in tails was quantified to indicate the degree of DNA damage. G) *γ*‐H2AX foci assays of SF295 and SHG140 cells treated with STELB and/or a 1 Gy dose of IR. The formation and resolution of *γ*‐H2AX foci were assessed using immunofluorescence. Scale bar, 20 µm. H) Quantification of the number of *γ*‐H2AX foci per nucleus at each time point. Representative images are shown. Graphs are shown as the mean ± SEM from three independent experiments; *P*‐values, two‐tailed unpaired Student's t‐test; *p < 0.05; **p < 0.01; ***p < 0.001; ****p < 0.0001.

To examine the effects of STELB on IR treatment of GBM, we pretreated GBM cells with STELB followed by irradiation with different doses of IR. STELB inhibited the viability of established human GBM cell lines SF295, U87, U251, and T98G cells as well as patient‐derived primary GBM cell lines DT001 and SHG140 in a dose‐dependent manner (**Figure** **1**A and Figure S1A, Supporting Information). Pretreatment with STELB significantly enhanced the cytotoxicity of IR consistently across multiple GBM cell lines (Figure 1B,C and Figure S1B, Supporting Information). Moreover, compared to each single‐agent treatment, the combination of STELB and IR significantly enhanced apoptosis (Figure 1D and Figure S1C, Supporting Information) and augmented the cleavage of Caspase‐3 and PARP (Figure 1E and Figure S1D, Supporting Information). Since IR produces cytotoxic effects mainly through direct induction of DNA damage, we suspected that STELB exerted a radiosensitizing effect by affecting the DNA damage response. To test this possibility, we first examined the extent of both single‐ and double‐strand DNA breaks induced by STELB and IR in SF295, SHG140, and U87 cells using alkaline comet assays. Indeed, pretreatment with STELB alone increased the extent of DNA damage in a dose‐dependent manner; when in combination with IR, STELB significantly increased the extent of DNA damage compared to either of them alone (Figure 1F and Figure S1E,F, Supporting Information). In addition, STELB did not induce apoptosis in GBM cells at 24 h, suggesting that the STELB‐induced accumulation of DNA damages is prior to apoptosis (Figure S1G,H, Supporting Information). We also investigated the effects on cell cycle of GBM cells after treated with different doses of STELB and found no significant changes (Figure S1I,J, Supporting Information). Since *γ*‐H2AX foci can detect double‐strand DNA breaks (DSBs),^[^ ^12^ ^]^ we next measured their formation in GBM cells to determine the kinetics of cellular DSBs. We found that GBM cells pretreated with STELB consistently displayed delayed resolution of IR‐induced *γ*‐H2AX foci in different GBM cell lines (Figure 1G,H and Figure S1K,L, Supporting Information). These results suggest that STELB enhances the sensitivity of GBM cells to IR by delaying DNA damage repair.

### 2.2 STELB Increases the Sensitivity of GBM Cells to TMZ In Vitro

**Figure 2:**
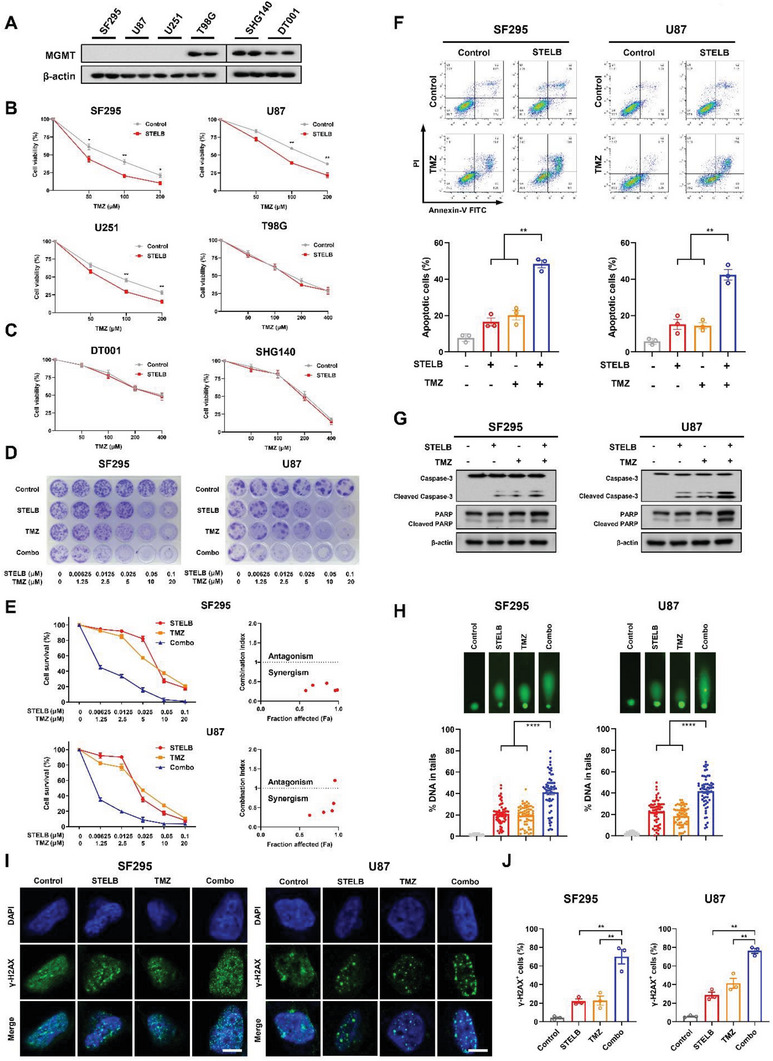
STELB sensitizes GBM cells to TMZ. A) Western blot analysis of MGMT status in different GBM cells. B,C) Cell viability assays for GBM cell lines pretreated with STELB for 24 h followed by exposure to TMZ at doses from 50 × 10^−6^ to 400 × 10^−6^ m for 72 h. D) Long‐term clonogenic assays for GBM cell lines treated with STELB and/or TMZ for 72 h, which were allowed to recover for 10–15 d and then subjected to crystal violet staining. E) Quantification of (D), the absorbance at 570 nm was measured after incubation with 1% SDS for 3 h. Cell survival (%) is expressed as % of the control; CIs were calculated with CalcuSyn. CI < 0.9 represents synergism, 0.9 < CI < 1.1 represents additivity, and CI > 1.1 represents antagonism. F) Apoptosis assays for GBM cells treated with STELB and/or 200 × 10^−6^ m TMZ for 72 h. Apoptosis was detected by Annexin V/PI staining. FACS quantification of the total apoptotic cell population, including Annexin V^+^/PI^−^ early apoptotic cells and Annexin V^+^/PI^+^ late apoptotic cells. G) Western blot analysis of Caspase‐3 and PARP in SF295 and U87 cells treated with STELB and/or TMZ for 48 h. H) Comet assays of SF295 and U87 cells treated with STELB and/or TMZ for 48 h. The % DNA in tails was quantified to indicate the degree of DNA damage. I) *γ*‐H2AX foci assays for SF295 and U87 cells were treated with STELB and/or TMZ for 48 h. The formation and resolution of *γ*‐H2AX foci were assessed using immunofluorescence. Scale bar, 20 µm. Representative images are shown. J) Quantification of the number of *γ*‐H2AX positive SF295 and U87 cells. Graphs are shown as the mean ± SEM from three independent experiments; P‐values, two‐tailed unpaired Student's t‐test; *p < 0.05; **p < 0.01; ***p < 0.001; ****p < 0.0001.

TMZ is a first‐line chemotherapeutic agent for GBM treatment that causes cancer cell death mainly by generating O^6^‐methylguanine lesions and then inducing DSBs.^[^ ^6c^ ^]^ As a DNA methyltransferase, O^6^‐methylguanine‐DNA methyltransferase (MGMT) directly removes the methyl group from the O^6^‐methylguanine lesions generated by TMZ and restores the normal guanine structure so that it cannot be converted into a DSB, thereby conferring resistance to TMZ.^[^ ^13^ ^]^ Therefore, the response of GBM cells to TMZ strongly depends on the status of MGMT. To investigate the effects of STELB on TMZ treatment, we performed cell viability assays on GBM cell lines with distinct MGMT status in combination with different concentrations of TMZ (**Figure** **2**A). We found that STELB significantly enhanced the inhibitory activity of TMZ on the cell viability of all three MGMT‐negative cell lines, SF295, U87, and U251 (Figure 2B) but not the MGMT‐positive cell lines (T98G, DT001, and SHG140) (Figure 2B,C and Figure S2A, Supporting Information). STELB did not affect MGMT‐expression in SHG140 cells, while slightly inhibited MGMT‐expression in T98G cells at 1 × 10^−6^ m (Figure S2B, Supporting Information), a concentration much higher than its anti‐proliferative IC_50_ (Figure S1A, Supporting Information), which was presumably due to the “off‐target” cytotoxic effect of STELB. These results suggest that STELB enhanced the sensitivity of GBM cells to TMZ by promoting TMZ‐induced O^6^‐methylguanine lesions and consequent DSBs. Using long‐term clonogenic assays, we further demonstrated a strong synergistic anti‐proliferative effect of STELB and TMZ (CI < 1) in all three MGMT‐negative GBM cell lines (Figure 2D,E and Figure S2C,D, Supporting Information). Consistently, our apoptosis assays showed greater‐than‐additive apoptosis‐inducing effects of STELB and TMZ (Figure 2F and Figure S2E, Supporting Information), accompanied by an enhanced cleavage of Caspase‐3 and PARP (Figure 2G). Moreover, we performed comet assays and *γ*‐H2AX foci assays and further confirmed a greater‐than‐additive effect of STELB and TMZ on the increase of the DNA damage in these cells (Figure 2H–J). Multiple timepoint analysis of *γ*‐H2AX foci showed that treatment with STELB in both SF295 and U87 cells resulted in persistently high levels of *γ*‐H2AX up to 24 h after TMZ treatment, in contrast to the control cells where *γ*‐H2AX could barely be detected (Figure S2F,G, Supporting Information). These results indicate that STELB enhances the efficacy of TMZ on GBM cells by delaying DNA damage repair.

### 2.3 STELB Increases the Sensitivity of GBM Tumor to TMZ In Vivo

**Figure 3:**
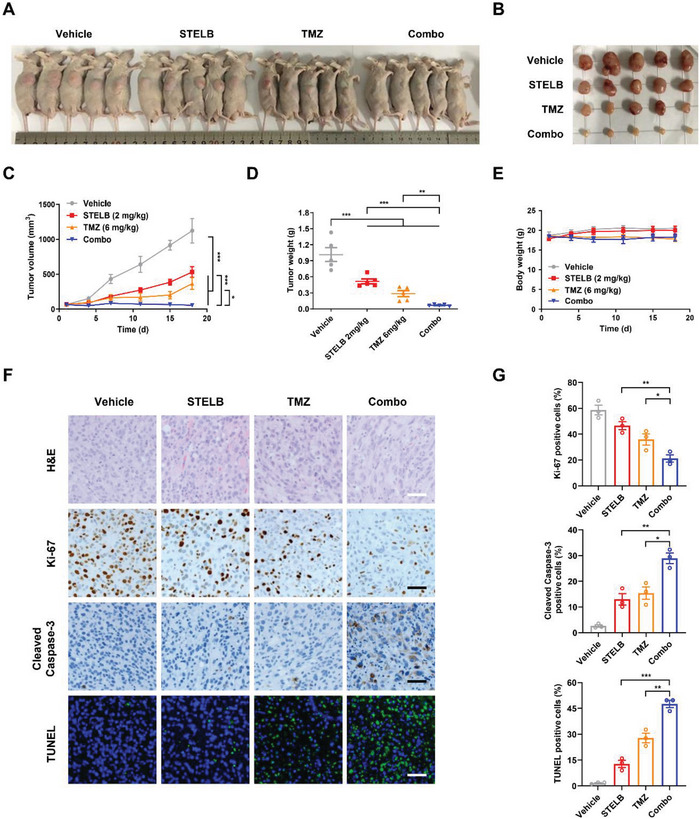
STELB sensitizes GBM to TMZ in a heterotopic nude mouse xenograft model Nude mice with subcutaneous U87 tumors were treated with vehicle, STELB (2 mg kg^−1^, IP), TMZ (6 mg kg^−1^, IP), or the STELB and TMZ combination for 18 d (*n* = 5). Images acquired after euthanasia are shown in (A). Excised tumors are shown in (B). Grid length, 20 mm. C) Tumor volumes, D) tumor weights, and E) body weights were measured every 3 d. F) H&E, Ki‐67, and Cleaved Caspase‐3 immunohistochemistry staining and TUNEL staining of tumor tissues from U87 mouse xenografts treated with STELB and/or TMZ. Scale bar: 50 µm. G) Statistical quantification of (F), each point represents the mean values of five images. *P*‐values, two‐tailed unpaired Student's t‐test; *p < 0.05; **p < 0.01; ***p < 0.001.

We next evaluated the effects of STELB, TMZ, and their combination on tumor growth in vivo. Using a subcutaneous xenograft tumor model of nude mice, we found that although administration of STELB and TMZ by themselves delayed tumor growth (**Figure** **3**A,B), their combination inhibited tumor growth more effectively than either alone and did not result in significant loss of body weight (Figure 3C–E). Immunohistochemistry analysis showed that compared to either STELB or TMZ, their combination i) inhibited the expression of the proliferation marker Ki‐67 more significantly and ii) enhanced apoptosis, as indicated by the increased cleavage of Caspase‐3 and number of TUNEL‐positive cells (Figure 3F,G). This result is consistent with our in vitro finding that STELB enhanced the cytotoxicity of TMZ. Taken together, our results demonstrated that administration of STELB and TMZ resulted in tumor growth inhibition greater than either drug alone, suggesting an improved efficacy and good tolerance of the combination therapy for clinical use.

### 2.4 STELB Preferentially Inhibits Homologous Recombination (HR) Repair in GBM Cells

**Figure 4:**
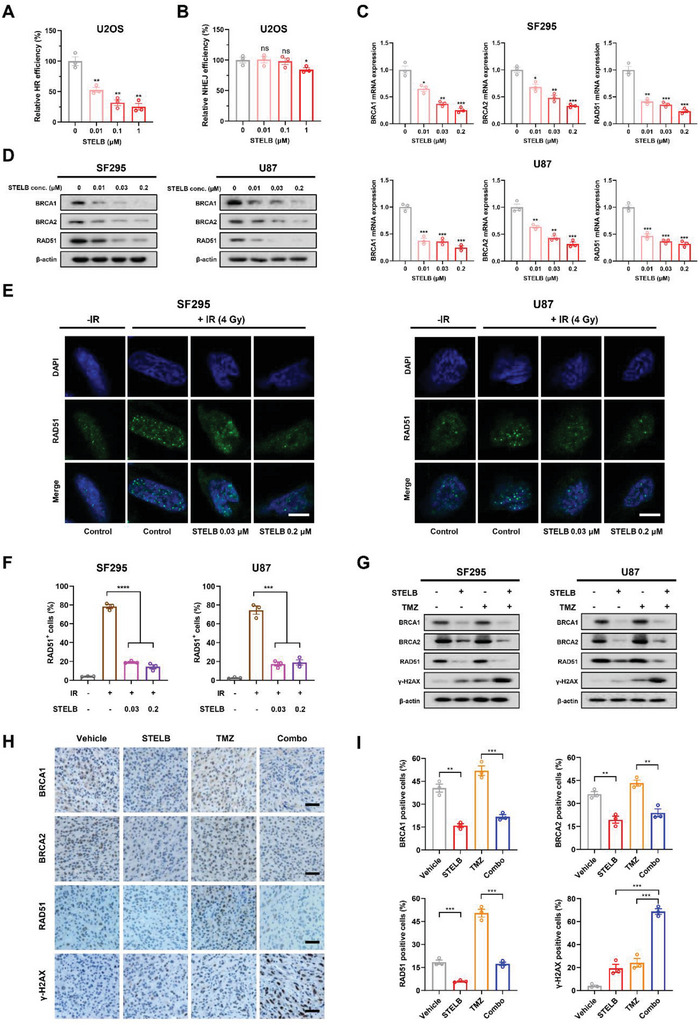
STELB impairs HR repair rather than NHEJ repair. A) HR reporter assays to detect the effect of STELB on HR repair efficiency in U2OS‐DR‐GFP cells. B) NHEJ reporter assays to detect the effect of STELB on NHEJ repair efficiency in U2OS‐EJ5‐GFP cells. C) The mRNA levels of key HR repair genes, BRCA1, BRCA2, and RAD51 in SF295 and U87 cells treated with STELB for 48 h, determined by qRT‐PCR. D) Western blot analysis of BRCA1, BRCA2, and RAD51 in SF295 and U87 cells treated with STELB for 48 h. E) RAD51 foci assays for SF295 and U87 cells treated with STELB and/or 4 Gy IR. After 48 h, the formation of RAD51 foci was assessed using immunofluorescence. Scale bar, 20 µm. Representative images are shown. F) Quantification of the number of RAD51‐positive SF295 and U87 cells treated with STELB, TMZ, or the combination of STELB and TMZ. G) Western blot analysis of BRCA1, BRCA2, RAD51, and *γ*‐H2AX in SF295 and U87 cells treated with STELB and/or 4 Gy IR for 48 h. H) BRCA1, BRCA2, RAD51, and *γ*‐H2AX immunohistochemistry staining of tumor tissues from U87 mouse xenografts treated with STELB and/or TMZ. Scale bar, 50 µm. I) Statistical quantification of (H), each point represents the average values of five images. Graphs are shown as the mean ± SEM from three independent experiments; *P*‐values, two‐tailed unpaired Student's t‐test; ns indicates not significant; *p < 0.05; **p < 0.01; ***p < 0.001; ****p < 0.0001.

Having established that STELB has the potential to improve the efficacy of IR and TMZ for patients with GBM, we sought to elucidate the mechanisms by which STELB affected DNA damage. We first examined the effect of STELB on DNA repair efficiency in osteosarcoma U2OS cells and GBM SF295 and U87 cells using HR and nonhomologous end joining (NHEJ) reporter assays. STELB reduced the HR repair efficiency, and the reduction positively correlated with the STELB concentration (**Figure** **4**A and Figure S3A, Supporting Information). In contrast, the efficiency of NHEJ repair did not show a significant decline at concentrations <1 × 10^−6^ m, which is >100‐fold greater than that of HR repair inhibition (Figure 4B and Figure S3B, Supporting Information). This result suggests that the sensitization of GBM to DNA‐damaging treatments is likely due to the inhibition of HR repair. We then assessed the effect of STELB on key molecules in the HR repair pathway. We found that the RNA levels of *BRCA1*, *BRCA2*, and *RAD51* were decreased in a dose‐dependent manner, consistent with their protein levels (Figure 4C,D). In contrast, the expression levels of key molecules in the NHEJ pathway,^[^ ^14^ ^]^ such as DNA‐PKcs, Ku70, and Ku80, were not affected by STELB at a concentration of <0.2 × 10^−6^ m, consistent with the results of the NHEJ reporter assays; similarly, XRCC1, which is mainly involved in the efficient repair of DNA single‐strand breaks (SSBs) induced by exposure to IR and alkylating agents,^[^ ^15^ ^]^ was not inhibited at concentrations where STELB effectively inhibited HR repair (Figure S3C, Supporting Information). We further evaluated the effect of STELB on the formation of RAD51 foci, a recombinase that is loaded by the BRCA1‐PALB2‐BRCA2 complex onto the single‐stranded DNA generated by cleavage in HR repair to form pre‐synaptic filaments,^[^ ^16^ ^]^ and therefore, can be used as a marker of HR repair activity. STELB strongly inhibited the formation of RAD51 foci in GBM cells after exposure to IR (Figure 4E,F), suggesting its interference with the process of HR repair. Western blot results revealed that TMZ upregulated the expression of BRCA1, BRCA2, and RAD51, suggesting the activation of HR repair. The addition of STELB blocked the increase of the above protein levels, and the combination increased the number of *γ*‐H2AX foci, suggesting an intensified DSB stress (Figure 4G). Consistent with the in vitro results, we found similar changes in the levels of these proteins in in vivo tumors (Figure 4H,I). Collectively, these results indicate that STELB preferentially inhibits HR repair, thereby exacerbating the accumulation of DSBs in GBM cells.

**Figure 5:**
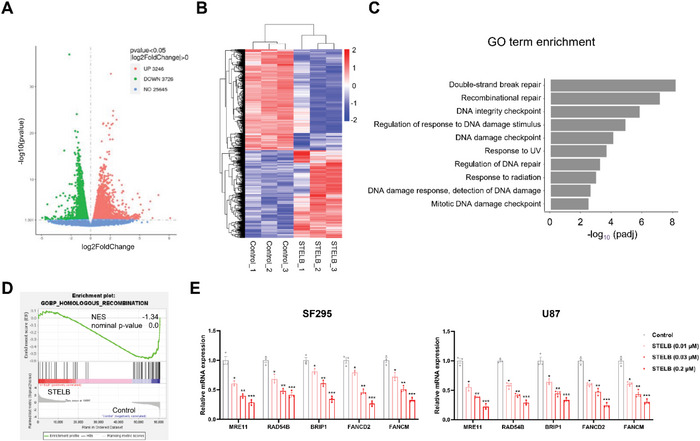
The transcriptome‐wide pattern of STELB treatment on DNA damage repair SF295 cells was treated with STELB (0.03 × 10^−6^ m) or DMSO (Control) for 24 h and subjected to RNA‐seq analysis. A) The volcano plot illustrates fold changes in gene expression in control cells compared to STELB‐treated cells. Significantly upregulated genes are shown in red, significantly downregulated genes are in green, and genes whose difference in expression was not statistically significant are shown in blue. Values are presented as the log_2_ of the tag counts. B) Hierarchical clustering shows significantly differentially expressed genes between control and STELB‐treated cells. C) Enrichment analysis for GO biological processes in control cells versus STELB‐treated cells. D) Gene set enrichment analysis of HR‐related genes in control cells versus STELB‐treated cells. Normalized enrichment score (NES), nominal *P* values are shown. E) qRT‐PCR analysis of SF295 and U87 cells treated with STELB to quantify representative DNA repair‐related genes detected in RNA‐seq data (mean ± SEM, *n* = 3). 18S rRNA was used for normalization. *p < 0.05; **p < 0.01; ***p < 0.001.

To confirm the effects of STELB on DNA damage repair more comprehensively, we performed RNA‐sequencing (RNA‐seq) to characterize transcriptome‐wide changes in SF295 cells following STELB treatment. We identified significant upregulation of 3246 genes and downregulation of 3726 genes (FDR < 0.05, **Figure** **5**A,B), and among them, the biological processes of DSB repair were the top enriched terms (GO analysis, *P* < 10^−8^, Figure 5C). We performed a gene set enrichment analysis (GSEA) and confirmed that HR repair‐related genes were significantly downregulated in STELB‐treated SF295 cells (Figure 5D). In terms of individual genes identified by differential analysis, the expression levels of BRCA1/2 and RAD51 were decreased, which were consistent with the qRT‐PCR and Western blot results (Figure 4C,D), as shown earlier. We also analyzed the expression of several other representative genes associated with HR repair (MRE11, RAD54B, BRIP1, FANCD2, and FANCM) and found a dose‐dependent reduction (Figure 5E). Thus, the unbiased RNA‐seq analysis further supports our proposed molecular mechanism that STELB sensitizes GBM cells to DNA‐damaging treatments through HR repair inhibition.

### 2.5 STELB Suppresses HR Repair‐Related Factors by Regulating PI3K Signaling

**Figure 6:**
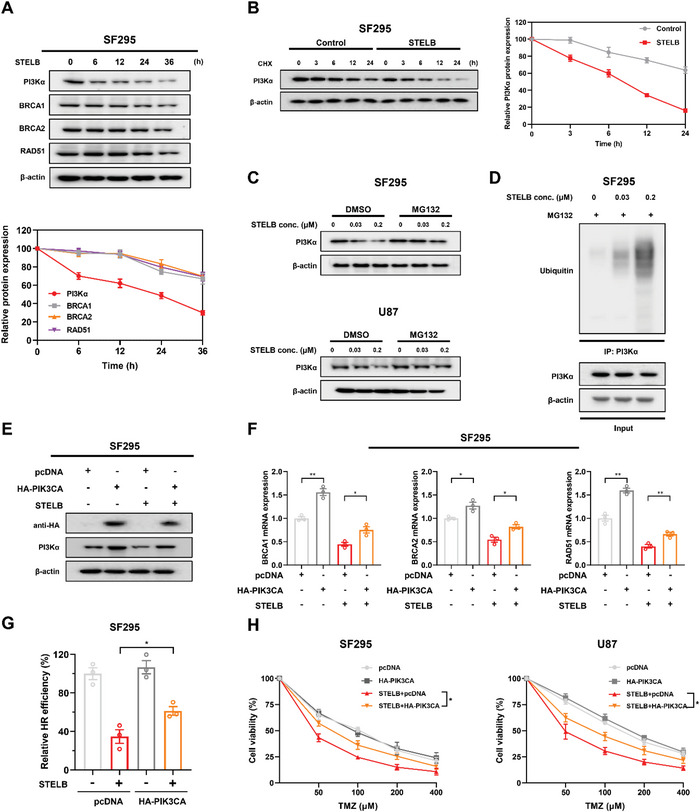
STELB promotes the ubiquitination and degradation of PI3K*α* to inhibit HR repair. A) Time‐course analysis of PI3K*α*, BRCA1/2, and RAD51 protein levels at the indicated time points in cell lysates from SF295 cells treated with STELB. B) Stability analysis of endogenous PI3K*α* protein in SF295 cells. Cells were treated with 20 × 10^−6^ m CHX for indicated durations and analyzed by Western blot. C) Western blot analysis of PI3K*α* proteins in total lysates of SF295 and U87 cells treated with increasing concentrations of STELB for 24 h followed by treatment with DMSO or MG132 for an additional 24 h. D) Ubiquitination assays of PI3K*α* upon treatment with STELB in SF295 cells. Ubiquitinated PI3K*α* was immunoprecipitated and subjected to Western blot analysis using an anti‐ubiquitin antibody. Cells were treated with MG132 before ubiquitination analysis. HA‐PIK3CA plasmid was transiently transfected into SF295 cells, followed by treatment with DMSO or STELB for 48 h. Western blot analysis was carried out to show overexpression of E) PI3K*α* (HA‐Tag mAb), qRT‐PCR, and HR reporter assays to measure the effect of PI3K*α* overexpression on F) the mRNA level of key HR repair factors and G) the HR repair efficiency. H) Cell viability assays for SF295 and U87 cells with transiently transfected HA‐PI3K*α* plasmid for PI3K*α* overexpression. TMZ or the combination of STELB and TMZ was added the next day, and cell viability was determined using CCK‐8 reagent 96 h later. Graphs are shown as the mean ± SEM from three independent experiments; *P*‐values, two‐tailed unpaired Student's t‐test. *p < 0.05; **p < 0.01.

To gain further mechanistic insights into the inhibitory effects of STELB on HR repair, we focused on the PI3K signaling pathway. This pathway plays a critical role in tumorigenesis and progression and has become important for targeted therapy;^[^ ^17^ ^]^ several studies have reported its regulatory role in the HR repair of DSBs.^[^ ^18^ ^]^ We first used an Adapta kinase assay to assess the effects of STELB on the kinase activity of the four PI3K isoforms and did not observe any inhibition at concentrations <10 × 10^−6^ m (Figure S4A, Supporting Information), which is 1000‐times the concentration at which STELB starts inhibiting the expression of HR repair‐related proteins (0.01 × 10^−6^ m). Therefore, PI3K is unlikely to be the direct target of STELB. Next, we used Western blots to examine the inhibition of the expression of PI3K and the phosphorylation of downstream molecules. We found that STELB exhibited strong inhibitory effects on the expression of the four PI3K isoforms and phosphorylation of Akt and mTOR in GBM cells (Figure S4B, Supporting Information). PI3K catalyzes the formation of PIP3 from PIP2, and the lipid phosphatase PTEN antagonizes PI3K by dephosphorylating PIP3 into PIP2.^[^ ^19^ ^]^ Interestingly, the expression of PTEN protein was not affected by STELB in PTEN wild‐type GBM cells, SF268 and LN229^[^ ^20^ ^]^ (Figure S4C, Supporting Information). To dissect the dynamic changes in PI3K and HR repair‐related proteins, we examined their expressions at different time points after STELB treatment. PI3K*α* markedly decreased in abundance by ≈50% at 6 h, while BRCA1, BRCA2, and RAD51 levels did not show a significant decrease until 24 h (**Figure** **6**A). To determine the role of PI3K in the expression of these proteins in GBM cells, we knocked down the PIK3CA gene in SF295 and U87 cells using siRNAs and found that the knockdown significantly reduced the expression of these three key components of HR repair at both the mRNA and protein levels (Figure S5A,B, Supporting Information). Pharmacological inhibition of PI3K yielded similar phenotypes (Figure S5C,D, Supporting Information).

We then examined how STELB reduced PI3K protein abundance in GBM cells. We did not observe a significant decrease in the mRNA expression of any of the four PI3K isoforms by qRT‐PCR (Figure S6A, Supporting Information), suggesting that STELB downregulated protein levels but not by reducing the mRNA levels of PI3K. We, therefore, speculated that STELB affects PI3K protein levels through post‐translational regulation. To test this hypothesis, we performed a half‐life analysis in SF295 cells using cycloheximide (CHX). We extracted total proteins at different time points with or without STELB treatment and examined the endogenous PI3K*α* protein levels by Western blot. Compared to the control group, STELB significantly shortened the half‐life of the PI3K*α* protein (Figure 6B). We observed similar findings when we transfected exogenous HA‐PIK3CA in 293T cells (Figure S6B, Supporting Information). Furthermore, the proteasome inhibitor MG132 effectively reversed the reduction in PI3K*α* caused by STELB (Figure 6C). We pulled down the PI3K*α* protein and found that despite basal ubiquitination, the addition of STELB greatly enhanced the ubiquitination level (Figure 6D). These results suggest that STELB reduces the abundance of PI3K protein by promoting the ubiquitination and degradation of PI3K, which subsequently inhibits the expression of HR repair‐related proteins. We further demonstrated that in GBM cells, HR repair efficiency decreased significantly after both genetic and pharmacological inhibition of PI3K (Figure S7A,B, Supporting Information); overexpression of PIK3CA partially reversed the decreases in mRNA expression of HR repair‐related molecules BRCA1/2 and RAD51 and the reduction in HR repair efficiency induced by STELB (Figure 6E–G). Moreover, overexpression of PIK3CA reduced the sensitivity of GBM cells to the combined administration of STELB and TMZ (Figure 6H). These results collectively indicate that STLEB impairs HR repair in GBM cells by increasing the ubiquitination and degradation of PI3K*α*, thus aggravating TMZ‐induced DNA damage.

### 2.6 STELB Has the Potential to Cross the Blood‐Brain Barrier (BBB) to Exert Anti‐GBM Effects

**Figure 7:**
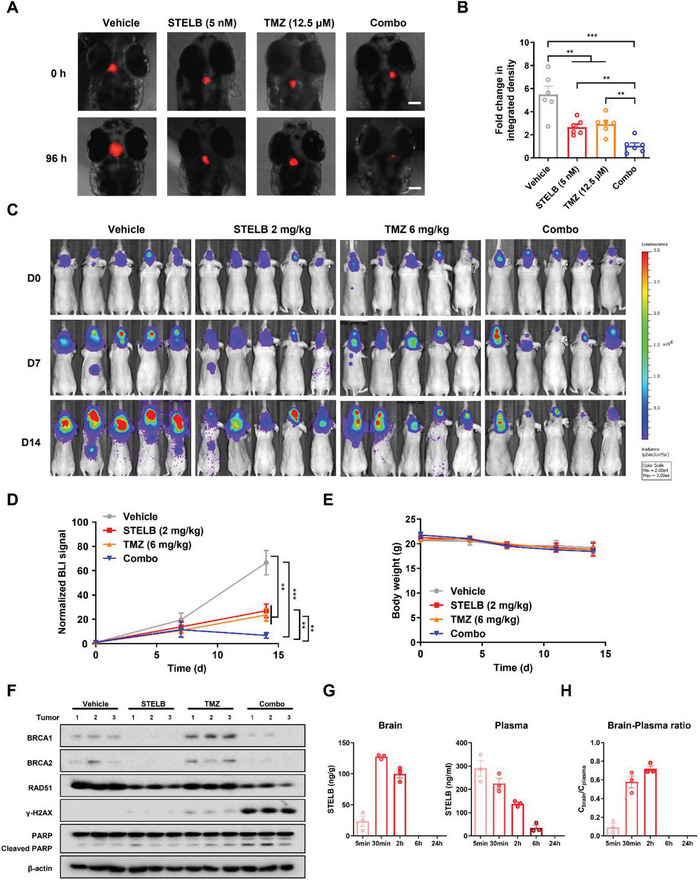
STELB sensitizes GBM to TMZ in orthotopic zebrafish and nude mouse xenograft models. A) Fluorescence image of embryos in the orthotopic zebrafish model. After transplantation of 50–100 U87‐RFP cells, the injected embryos were transferred to a 96‐well plate containing drug and incubated for 96 h. The embryos were imaged under a fluorescence microscope to evaluate tumor growth. Scale bar, 100 µm. B) The fluorescence intensity of different groups, as determined at experimental endpoint (day 4) and expressed as fold change in integrated density. A student's t‐test was used to compare the effect of each two groups. C) Bioluminescence images of the nude mouse xenograft model mice. Mice were intracranially inoculated with U87‐Luc cells. The localization and intensity of luciferase expression were monitored by in vivo bioluminescence imaging. Following a single dose of STELB (2 mg kg^−1^, IP), TMZ (6 mg kg^−1^, IP), or combination treatment of STELB and TMZ for 14 d (*n* = 5), animals were assessed using a bioluminescence imaging system. Representative bioluminescent images acquired at the indicated time points are shown. D) The relative radiance on days 0, 7, and 14, which represents tumor size, was determined using the imaging system. Values on day 14 were used for statistical analysis of the difference between any two groups. *p < 0.05; **p < 0.01; ***p < 0.001. E) Bodyweight curves during the 14 days of treatment. (F) Western blot analysis of BRCA1, BRCA2, RAD51, *γ*‐H2AX, and PARP in tumor lysates. Three representative samples were selected from each group. G) STELB concentrations in blood and brain, analyzed by LC‐MS after one injection of 3 mg kg^−1^. H) The brain‐to‐plasma ratio across different time points.

The capability to cross the BBB is a requisite for drug development aiming to treat intracranial tumors such as GBM.^[^ ^21^ ^]^ This requirement is often a “death sentence” in clinical practice for the vast majority of lead compounds that are very effective for GBM treatment in vitro.^[^ ^22^ ^]^ To preliminarily assess the potential of STELB to cross the BBB, we first transplanted fluorescently labeled U87 cells into the zebrafish cranium to allow accurate characterization of the tumor growth state. We found that both STELB and TMZ administered as single agents inhibited tumor growth in the zebrafish cranium to some extent, but their combination exhibited a much stronger inhibitory effect (**Figure** **7**A,B). Next, we established a U87 orthotopic xenograft tumor model in nude mice to examine the anti‐GBM effects. Both drugs significantly inhibited tumor growth compared to the control, and this inhibition was more prominent for the combination. Importantly, the combined STELB/TMZ administration induced regression of orthotopic tumor (Figure 7C,D), while no significant change was observed in body weight (Figure 7E), suggesting favorable anti‐GBM efficacy and safety. Western blot assays of tumor lysates demonstrated that DNA damage and apoptosis were induced only modestly by STELB or TMZ when administered alone but were significantly enhanced upon coadministration, as evidenced by significant decreases in BRCA1, BRCA2, and RAD51 levels and an increase in *γ*‐H2AX level and PARP cleavage (Figure 7F). In addition, the expression levels of DNA‐PKcs, Ku70, and Ku80 were not affected by STELB, consistent with the in vitro data (Figure S8, Supporting Information). We also examined the distribution and preliminary pharmacokinetics of STELB in mice. STELB rapidly entered the mouse brain and reached a peak concentration in ≈30 min, and the brain/plasma concentration ratio reached a peak at 2 h (Figure 7G,H). These results show preliminary evidence of the potential of STELB as an effective therapeutic to cross BBB to exert anti‐tumor effects.

**Figure 8:**
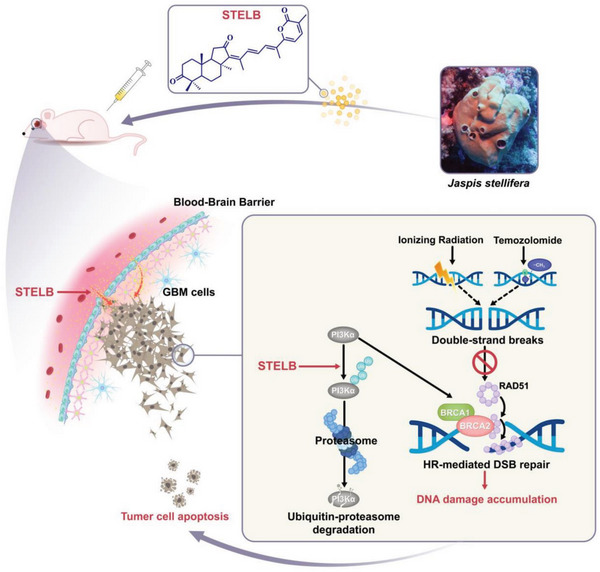
Schematic summary of the main findings in this study.

In summary, our results show that STELB has the potential to cross the BBB to promote the degradation of PI3K*α* through the ubiquitination‐proteasome pathway, thereby reducing the expression of key components of the HR repair pathway, such as BRCA1, BRCA2, and RAD51. Thus, through intensifying HRD, STELB impairs the repair of DSBs caused by IR or TMZ, leads to exacerbation of DNA damage, and ultimately increases GBM cell death (**Figure** **8**).

## 3 Discussion

The development of novel therapeutic approaches for GBM has always been a great challenge. The highly heterogeneous nature of GBM adds complexity to the development of effective therapies, and the intracranial location of the lesions makes most drugs inaccessible.^[^ ^22b^ ^]^ In addition to surgery, the current treatment of GBM relies on more broad‐spectrum anticancer modalities—radiotherapy and chemotherapy based on the alkylating agent TMZ, but the inherent or secondary resistance to these therapies represents a huge clinical challenge worldwide.^[^ ^23^ ^]^ Despite tremendous efforts to address chemo‐ and radiotherapy resistance through combination therapy, only a handful of regimens have moved to the clinical trial stage, with little success.^[^ ^2^, ^3^ ^]^ In this study, for the first time, we show that STELB has the potential to cross the BBB to sensitize GBM cells against radiotherapy and TMZ chemotherapy by inhibiting HR repair, which is at least partially mediated by promoting ubiquitination and degradation of PI3K. Importantly, we show preliminary evidence that STELB was able to cross the BBB rapidly and inhibit GBM growth in situ in two animal models, which is an essential property for effective GBM therapy. Collectively, our results provide strong evidence that STELB is a promising drug candidate in combination with first‐line radiation or chemotherapy for improving the clinical outcome of patients with GBM.

Given the unknown mode of action of STELB, we started with a phenotypic discovery approach, which has long been a proven drug development strategy. A decade ago, Swinney and Anthony published a landmark study showing that among 183 small molecule drugs approved in all therapeutic areas between 1999 and 2008, 58 (32%) were discovered using a phenotype‐based approach.^[^ ^24^ ^]^ For example, Nakajima et al. identified FR901228 as a novel histone deacetylase inhibitor by screening for microbial metabolites that induced transcriptional activation of the SV40 promoter;^[^ ^25^ ^]^ halichondrin B, a large polyether macrolide found in sponges, induces G2‐M cell cycle arrest and disrupts the organization of the mitotic spindle, and its synthetic derivative eribulin was later approved as a drug targeting microtubules.^[^ ^26^ ^]^ Our study represents another successful example of this approach.

We not only established the ability of STELB as a sensitizer for IR or TMZ in the treatment of GBM but also showed that these effects are through the inhibition of HR. IR and TMZ kill GBM cells primarily by inducing DNA damage, specifically DSBs. DSBs are the most lethal type of DNA damage but can be repaired by both HR and NHEJ. HR uses sister chromatids with sequence homology as the template for repair, has a high degree of fidelity, and is the only way to correctly repair DSBs. NHEJ does not require homology between DNAs and directly connects the broken ends; however, with less accuracy, which can lead to gene mutations or chromosomal rearrangements, and even when the repair is completed, the cell still has a greater chance to die eventually.^[^ ^27^ ^]^ We show that STELB preferentially inhibited HR over NHEJ, with three orders of magnitude difference in selectivity. Moreover, RNA‐seq‐based analysis suggests a global impact of STELB on DNA damage repair, further validating our proposed drug mechanisms. Since one leading cause underlying IR and TMZ resistance is the upregulated DNA damage response, STELB thus may prevent such induced resistance. Furthermore, our results revealed that the effect of STELB on HR is, at least in part, by a reduction in PI3K protein abundance; and that STELB promotes the degradation of PI3K*α* through the ubiquitination‐proteasome pathway rather than affecting its mRNA expression or kinase activity, consistent with the literature that PI3K is regulated by polyubiquitination.^[^ ^28^ ^]^ Two recent studies demonstrated that PI3K inhibition could induce HRD in breast cancer by decreasing BRCA1/2 expression and RAD51 foci formation,^[^ ^18a,b^ ^]^ laying a mechanistic basis for several clinical trials combining PI3K inhibitors with PARP inhibitors or other DNA damaging agents.^[^ ^29^ ^]^ Given that the PI3K pathway is aberrantly activated in 56–75% of patients with GBM, the mechanism of action of STELB is of particular interest for this disease. Further efforts, including the study of BBB penetrance of STELB in greater depth and clinical trials, should be made to assess the utility of STELB (or analogs) in the treatment of patients with GBM.

## 4 Experimental Section

### Reagents and Antibodies

STELB was isolated from *Jaspis stellifera* and structurally identified it as described previously.^[^ ^10a^ ^]^ TMZ, BKM120, and ZSTK474 were purchased from Selleck Chemicals. The following antibodies were used: Caspase‐3 (Cell Signaling Technology, 9662), Cleaved Caspase‐3 (Cell Signaling Technology, 9664), PARP (Cell Signaling Technology, 9532), MGMT (Cell Signaling Technology, 86039), PTEN (Cell Signaling Technology, 9188), PI3K*α* (Cell Signaling Technology, 4249), PI3K*β* (Cell Signaling Technology, 3011), PI3K*γ* (Cell Signaling Technology, 5405), PI3K*δ* (Cell Signaling Technology, 34050), phospho‐Akt (Thr308, Cell Signaling Technology, 13038), phospho‐Akt (Ser473, Cell Signaling Technology, 4060), phospho‐mTOR (Ser2448, Cell Signaling Technology, 5536), DNA‐PKcs (Cell Signaling Technology, 38168), Ku70 (Cell Signaling Technology, 4588), Ku80 (Cell Signaling Technology, 2180), XRCC1 (Cell Signaling Technology, 2735), *γ*‐H2AX (Abcam, ab81299), BRCA1 (Cell Signaling Technology, 14823, for Western blot), BRCA1 (Abcam, ab16780, for IHC), BRCA2 (Abcam, ab216972), RAD51 (Abcam, 133534), Ki‐67 (Cell Signaling Technology, 9027), HA‐Tag (Cell Signaling Technology, 3724), *β*‐actin (Cell Signaling Technology, 4970), anti‐rabbit IgG, HRP‐linked antibody (Cell Signaling Technology, 7074), and anti‐rabbit IgG (H+L), F(ab')_2_ Fragment (Alexa Fluor 488 Conjugate, Cell Signaling Technology, 4412).

### Cell Culture and Transfection

U87, U251, and LN229 cells were purchased from the Cell Bank of the Chinese Academy of Sciences (Shanghai, China). T98G, U2OS, and 293T cells were obtained from the American Type Culture Collection (Manassas, VA, USA). SF295 and SF268 cells were provided by the National Cancer Institute (NCI, USA). SHG140 and DT001 are primary GBM cells isolated from fresh tumor tissues, provided by Dr. Zhennan Tao of Department of Neurosurgery & Brain and Nerve Research Laboratory, The First Affiliated Hospital of Soochow University, and Dr. Yuxiang Dai of Department of Neurosurgery, the Affiliated Drum Tower Hospital, School of Medicine, Nanjing University, respectively. Cells were cultured in Dulbecco's modified Eagle medium (DMEM) or Roswell Park Memorial Institute 1640 medium (RPMI 1640) supplemented with 10% fetal bovine serum (FBS, Gibco), penicillin (100 U mL^−1^), and streptomycin (100 µg mL^−1^) in a humidified incubator with an atmosphere containing 5% CO_2_ at 37 °C. Cells were passaged routinely every 2–3 d and maintained for a maximum of 20 passages of subculture. To overexpress PIK3CA, cells were transiently transfected with HA‐PIK3CA (Hanbio Biotechnology Co. Ltd) using Lipofectamine 3000 (Life Technologies).

### Cell Viability Assay

Cell viability was measured by CCK‐8 assay as previously described.^[^ ^11^ ^]^ Cells were seeded at a density of 5 × 10^3^ cells mL^−1^ in a volume of 200 µL per well in 96‐well plates. The next day, cells were treated with DMSO or the indicated concentrations of STELB and/or TMZ. After 48 h or 96 h, 10 µL of CCK‐8 (Beyotime) was added to each well. After another 2 h incubation at 37 °C, the absorbance was measured at 450 nm using an iMark microplate reader (Bio‐Rad).

### IR and Clonogenic Survival Assay

Irradiation was conducted using an X‐RAD 320 cabinet irradiator (Precision X‐Ray) at doses from 0 to 6 Gy and a dose rate of 250 MU min^−1^. To determine the effects of STELB on IR‐induced cell death, 500–1000 cells per well were seeded in triplicate in six‐well plates and incubated overnight. After pretreatment with different concentrations of STELB or DMSO for 24 h, the cells were irradiated. Fresh drug‐free medium was added to the plates and replaced every 3 d. After incubation for 10–15 d, cells were stained with 0.25% crystal violet (Sigma‐Aldrich). The colonies in each well were visually quantified. The surviving fraction was calculated using GraphPad Prism 8 and was normalized to that of unirradiated control cells. To determine the synergistic effects of STELB and TMZ, 100–400 cells were seeded in 24‐well plates and incubated overnight and then treated with both drugs simultaneously for 72 h. Normal medium was added to the plates and replaced every 3 d. After incubation for 10–15 d, cells were stained with 0.25% crystal violet. Plates were scanned to capture images. Cells were lysed in 1% sodium dodecyl sulfate (SDS), and optical density (OD) at 570 nm was determined using an iMark microplate reader (Bio‐Rad).

### Apoptosis Assay

Apoptosis analysis was performed as previously described.^[^ ^30^ ^]^ Briefly, cells were treated with STELB and/or IR or TMZ. After harvesting, the cells were resuspended in 100 µL of binding buffer and incubated with Annexin V/PI solution (Annexin V‐FITC/PI Apoptosis Detection Kit, BD Biosciences) in the dark for 15 min. Samples were acquired on a FACS Verse Flow Cytometer (BD Biosciences) and analyzed using FlowJo Software (BD Life Sciences).

### Cell Cycle Analysis

Cells were seeded into six‐well plates and treated with STELB. After 48 h of incubation, cells were collected, suspended in PBS, and fixed in 75% ethanol at 4 °C overnight. Next, cells were washed and resuspended in PBS containing 50 µg mL^−1^ PI and 100 µg mL^−1^ RNase (Solarbio). All samples were analyzed on a FACS Verse Flow Cytometer (BD Biosciences).

### Alkaline Comet Assay

Alkaline comet assays were performed as previously described.^[^ ^31^ ^]^ After treatment with STELB and/or IR or TMZ, cells (1  ×  10^5^ per mL) were collected and mixed with low melting point agarose at a ratio of 1:10 (v/v), and 50 µL of the cell suspension was immediately added onto comet slides. The slides were then incubated at 4 °C for 10 min, immersed in lysis solution for 30 min, and in alkaline unwinding solution for 20 min in the dark. Following electrophoresis, the cells were stained with SYBR Gold (Invitrogen). The quantification of tail DNA was performed with CASP software (CaspLab).

### Immunofluorescence Staining

Immunofluorescence staining was performed as previously described.^[^ ^32^ ^]^ Cells were seeded onto coverslips in 24‐well plates at a density of 5 × 10^3^ cells per well in 1 mL of medium. After 24 h, cells were treated with STELB and/or IR or TMZ for indicated durations. Cells were fixed in 4% paraformaldehyde and permeabilized with 0.2% Triton X‐100 in PBS. The cells were then blocked in 5% donkey serum in the presence of 0.1% Triton X‐100 and stained with the *γ*‐H2AX or RAD51 primary antibody. Cells were washed three times with PBS, and the secondary antibody coupled to Alexa Fluor 488 was added and incubated for 1 h at room temperature. After being rinsed and washed three times with PBS, slides were mounted with VECTASHIELD mounting medium (Vector Laboratories) containing DAPI. Cells were observed using a BX51 fluorescence microscope (Olympus).

### siRNA Knockdown

siRNA knockdown of PIK3CA was performed as previously described.^[^ ^33^ ^]^ siRNA for PIK3CA (5'‐CUGAGAAAAUGAAAGCUCACUCUTT‐3') was purchased from Sigma‐Aldrich. Briefly, cells were plated in six‐well plates and transfected with 30 × 10^−9^ m of PIK3CA‐targeting or nontargeting siRNA (siNT) using Lipofectamine 3000 transfection reagent (Invitrogen) according to the manufacturer's protocol. Cells were collected 72 h post‐transfection for qRT‐PCR analysis, Western blot analysis, and HR reporter assay.

### qRT‐PCR

qRT‐PCR was performed as previously described.^[^ ^34^ ^]^ RNA was isolated using an RNeasy Mini kit (Qiagen) and used to synthesize complementary DNA (cDNA) using a cDNA Synthesis Kit (GenStar), and RT‐PCR was performed with aliquots of cDNA samples mixed with SYBR Green Master Mix (Applied Biosystems). Reactions were performed in triplicate. The fold differences in transcripts were calculated using the ΔΔCt method, and 18S rRNA was used as a control to normalize RNA expression (Table S1, Supporting Information shows the primers used).

### Western Blots

Western blots were performed as previously described.^[^ ^35^ ^]^ Cells were seeded in six‐well plates at a density of 2 × 10^5^ cells per well in 2 mL of medium. After 24 h, the cells in each well were treated with STELB and/or IR or TMZ. Cells were lysed, and total proteins were harvested. Equal amounts of protein (20–50 µg) were separated using 8% or 12% SDS‐PAGE and transferred to polyvinylidene difluoride membranes (Bio‐Rad). After blocking in 5% nonfat dry milk, the membranes were incubated with appropriate primary antibodies overnight at 4 °C, washed, and incubated with respective HRP‐conjugated secondary antibodies for 1 h at room temperature. The signals were detected using a ChemiDoc XRS+ System (Bio‐Rad) after exposure to chemiluminescence reagents (Bio‐Rad), and *β*‐actin served as the loading control.

### HR Reporter Assay

HR efficiency was evaluated as previously described.^[^ ^36^ ^]^ U2OS cells containing a single copy of the HR repair reporter substrate DR‐GFP integrated at random sites were transfected with an I‐SceI‐expression plasmid. For SF295 and U87 cells, the HR repair reporter substrate pDR‐GFP plasmid and the I‐SceI‐expression plasmids/empty vectors were cotransfected into cells using Lipofectamine 3000 (Invitrogen). GFP‐expressing plasmid (pEGFP‐C1) was used as a transfection efficiency control. STELB or BKM120 was added 6 h after the transfection. After 48 h, GFP‐positive cells were detected on a FACS Verse flow cytometer (BD Biosciences).

### NHEJ Reporter Assay

NHEJ efficiency was evaluated as previously described.^[^ ^37^ ^]^ In U2OS‐EJ5‐GFP cells, an I‐SceI‐induced DSB was generated within a chromosomally integrated inactive GFP cassette, and the GFP cassette was restored when the DSB was repaired by NHEJ. I‐SceI‐expression constructs or empty vectors were transfected into cells. For SF295 and U87 cells, EJ5‐GFP plasmid and the I‐SceI‐expression plasmids/empty vectors were cotransfected into cells using Lipofectamine 3000 (Invitrogen). GFP‐expressing plasmid (pEGFP‐C1) was used as a transfection efficiency control. STELB was added 6 h after transfection. GFP‐positive cells were quantified by flow cytometry (BD Biosciences) 48 h after transfection.

### Immunoprecipitation

Immunoprecipitation was performed as previously described.^[^ ^38^ ^]^ Cell lysates were prepared using lysis buffer supplemented with phosphatase inhibitors and complete protease inhibitors (Roche). Then, 500 µL of cell lysate was incubated with appropriate antibodies overnight at 4 °C. Prewashed protein A/G agarose beads (Invitrogen) were added to Ab‐lysate mixtures and incubated on a rotator for 3 h at 4 °C. The beads were washed three times with lysis buffer and centrifuged for 10 min at 5000 × *g*. Proteins were eluted from the beads with 20 µL of loading buffer and subjected to Western blot analysis as described above.

### In Vitro Kinase Assay

An in vitro kinase assay was performed as previously described^[^ ^10b^ ^]^ using the Adapta Universal Kinase Assay kit (Invitrogen) following the manufacturer's protocols with modifications. A dilution of STELB, ZSTK474 (positive control), or DMSO was prepared and added to appropriate wells of the 384‐well plate. Then, the optimized kinase solutions (PIK3CA/PIK3R1, PIK3CB/PIK3R1, PIK3CG, and PIK3CD/PIK3R1) were added to each well of the assay plate. Next, ATP and PIP2:PS lipid kinase substrate were added and incubated for 1 h at room temperature. A detection solution consisting of 30 × 10^−3^ m EDTA, 6 × 10^−9^ m Eu‐labeled anti‐ADP antibody, and 3 × Alexa Fluor 647 ADP tracer was added to all the wells and allowed to equilibrate for 30 min at room temperature. After incubation, the fluorescence of each sample was measured using a Multi‐Mode Microplate Reader (Spark, Tecan).

### Heterotopic Nude Mouse Xenograft Model

All mice care and experimental protocols were approved by the Ethical Committee of Tianjin Medical University (permit number: SYXK 2019‐0004). To generate a murine subcutaneous tumor model, U87 cells were subcutaneously injected into the right lateral flank of 4‐ to 5‐week‐old male BALB/c nude mice (Vital River Laboratory Animal Technology Company, Beijing, China). When tumors reached a volume of 800–1000 mm^3^, they were excised, diced into 2 mm × 2 mm × 2 mm pieces, and implanted into the right flanks of 20 BALB/c nude mice. Tumors were allowed to grow to a volume of 100 mm^3^, and then the animals were randomly divided into four groups. Each group of five mice was treated with either vehicle, STELB (2 mg kg^−1^, IP), TMZ (6 mg kg^−1^, IP), or STELB and TMZ (using the same doses as the single agent; simultaneous administration). Tumor size was measured every 3 d until the endpoint. The tumor volume was calculated using the following formula: (length × width^2^)/2. At the end of the 18 d experimental period, mice were euthanized by overdosing on pentobarbital sodium, and the tumors were removed. Half of each tumor tissue was formalin‐fixed and paraffin‐embedded for histological analysis, and the other half was snap‐frozen in liquid nitrogen for Western blot.

### H&E, TUNEL, and Immunohistochemical (IHC) Staining

H&E and IHC staining were performed as previously described.^[^ ^18c^ ^]^ H&E staining was used to detect pathological changes in morphology. Apoptotic cells in tumor tissues were stained using a TUNEL Apoptosis Detection Kit (Beyotime). For histological analysis, formalin‐fixed, paraffin‐embedded tumors were sectioned, and slides were deparaffinized using xylene (Thermo Fisher). Endogenous peroxidases were quenched with 3% hydrogen peroxide in methanol. Staining was performed using antibodies against BRCA1 (1: 100), BRCA2 (1: 100), RAD51 (1: 50), *γ*‐H2AX (1: 50), Cleaved Caspase‐3 (1:100), or Ki‐67 (1:500). Counterstaining was performed with Mayer's hematoxylin (Dako). Slides were observed under an Olympus CX21 microscope and scanned with a high‐resolution digital slide scanner (Pannoramic 250, 3DHistech) to capture images.

### Orthotopic Zebrafish Xenograft Model

Xenotransplantation of human GBM U87 cells and proliferation assessment were performed as previously described.^[^ ^18c^ ^]^ Wild‐type AB zebrafish (*Danio rerio*) were maintained and reared under a 14 h light, 10 h dark cycle at 28 °C in a controlled multi‐tank recirculating system. Embryos were collected and incubated in reconstituted water (60 µg mL^−1^ sea salt in RO water with 1 ppm methylene blue). At 48 h postfertilization, embryos were anesthetized using 1.2 ×10^−3^ m tricaine and moved onto a modified agarose gel mold for tumor cell microinjection. A total of 50–100 U87‐RFP cells suspended in 5 nL of serum‐free culture medium were injected into the brains of zebrafish larvae using a pneumatic pico‐pump injector. After cell implantation, injected embryos were screened and separately transferred to a 48‐well plate containing drugs (vehicle, STELB (5 × 10^−9^ m), TMZ (12.5 × 10^−6^ m), STELB and TMZ (simultaneous administration)) in 2 mL of E3 media and incubated at 32 °C for 4 d. Xenografts were observed under an inverted microscope (IX71, Olympus) every two days. The fluorescence intensity of xenografts was quantified with ImageJ software.

### Orthotopic Nude Mouse Xenograft Model

The murine intracranial tumor model was generated as previously described.^[^ ^18c^ ^]^ U87‐Luc cells (2.0 × 10^5^ cells per mouse) were injected into the right striatum of 4‐week‐old male BALB/c nude mice. Ten days after injection (day 0), tumors were measured using an IVIS luminescent imaging system (IVIS Spectrum, PerkinElmer) and randomly assigned to the following four treatment groups: vehicle, STELB (2 mg kg^−1^), TMZ (6 mg kg^−1^), STELB and TMZ (with the same doses as every single agent; simultaneous administration). The treatments were given daily. On days 7 and 14, animals were examined by luminescence imaging. The mice were euthanized on day 14 to collect tumor samples. Data were analyzed using in vivo imaging software (Living Image 4.5.2, PerkinElmer) and normalized to the initial post‐injection signal on day 0.

### Analysis of STELB Distribution by LC‐MS

Nude mice (*n* = 3 at each time point) were injected intraperitoneally with STELB at a loading dose of 3 mg kg^−1^, and brain and blood samples were harvested at 5, 30, 120, 360, and 1440 min after injection. Tissue samples were quantitatively homogenized in 10% methanol. The homogenates were precipitated with acetonitrile and immediately analyzed on SHIMAZU LC‐30AD LCMS‐8060 with a CAPCELL PAK MG II C18 chromatographic column (2.0 mm × 100 mm, 3.0 µm) and a mobile phase (acetonitrile–water with 0.1% formic acid: acetonitrile). Ionization was conducted in positive mode, and the m/z transitions were 463 for STELB. The stock solution of STELB was prepared by dissolving the drug in acetonitrile and further diluted to obtain the standard solutions ranging from 1 ng mL^−1^ to 1 µg mL^−1^ for the validation measurement. The content of STELB was determined by comparing to the calibration line of the spiked samples.

### RNA‐Seq Profiling and Analysis

Cells were treated with STELB (0.03 × 10^−6^ m) or DMSO for 24 h, and total RNA was isolated using TRIzol reagent. RNA concentrations were quantified using a NanoDrop Spectrophotometer (Thermo Fisher), and RNA integrity was assessed using the RNA Nano 6000 Assay Kit on a Bioanalyzer 2100 system (Agilent Technologies). Only samples with RIN values of > 6.0 were used for experiments. A complementary DNA library was prepared, and sequencing was performed according to the Illumina standard protocol by Beijing Novel Bioinformatics Co., Ltd. (https://en.novogene.com/). Specifically, cDNA libraries were prepared using an Illumina NEBNext Ultra RNA Library Prep Kit. After cluster generation, the library preparations were sequenced on an Illumina NovaSeq 6000 platform, and 150 bp paired‐end reads were generated. For the data analysis, raw data (raw reads) in fastq format were first processed through in‐house Perl scripts. Clean reads were obtained by removing reads containing adapters, poly Ns, and low‐quality reads from raw data. Reference genome and gene model annotation files were downloaded from the genome website directly. The index of the reference genome was built using Hisat2 v2.0.5, and paired‐end clean reads were aligned to the reference genome using Hisat2 v2.0.5. Mapped reads of each sample were assembled using StringTie (v1.3.3b) in a reference‐based approach. Feature Counts v1.5.0‐p3 was used to quantify the read numbers mapped to each gene. Then, the FPKM of each gene was calculated based on the length of the gene and the read count mapped to the gene. Differential expression analysis of two groups was performed using the edgeR package (3.12.1). The resulting *P*‐values were adjusted using the Benjamini‐Hochberg method for controlling the false discovery rate. Genes with an adjusted P‐value < 0.05 were considered statistically differentially expressed. Gene Ontology (GO) analysis was performed with the cluster Profiler R package. The hierarchical clustering heat map was generated with the ggplot library. Gene Set Enrichment Analysis (GSEA) was performed using the software GSEA v2.2.2 (www.broadinstitute.org/gsea).

### Statistical Analysis

Data from three independent experiments are presented and expressed as mean ± SEM. Unpaired, two‐tailed Student's t‐tests were used for two‐group comparisons. ANOVA with Bonferroni's correction was used to compare multiple groups. A *P*‐value < 0.05 was considered statistically significant. Drug interactions were assessed as CIs, which were calculated with the CalcuSyn software program (Version 2.1, Biosoft). CI < 0.9 represents synergism, 0.9 < CI < 1.1 represents additivity, and CI > 1.1 represents antagonism. Before statistical analysis, variations within each group and the assumptions of the tests were assessed.

## Conflict of Interest

The authors declare no conflict of interest.

## Author Contributions

X.P., S.Z., Y.W., Z.Z., Z.Y., and Z.Z. performed the experiments. L.Z., Z.‐S.C., F.X.C., M.E., F.S., and F.W. analyzed the data. X.P., R.W., H.L., H.W.L., and D.K. designed the experiments. X.P., R.W., and D.K. wrote the main manuscript. Z.S.C., H.L., H.W.L. and D.K. revised the manuscript. All authors reviewed the manuscript.

## Supporting information

Supporting Information

## Data Availability

The data that support the findings of this study are available from the corresponding author upon reasonable request.
