# Supplementary material for: Stellettin B Sensitizes Glioblastoma to DNA‐Damaging Treatments by Suppressing PI3K‐Mediated Homologous Recombination Repair
Source: Adv Sci (Weinh). 2022 Dec 1;10(3):2205529. doi: 10.1002/advs.202205529 (PMC9875605; doi:10.1002/advs.202205529)
Supplement: Supplementary file 1 — Supporting Information [file ADVS-10-2205529-s001.pdf]

ADVANCED SCIENCE
Open Access

Supporting Information

for *Adv. Sci.*, DOI 10.1002/advs.202205529

Stellettin B Sensitizes Glioblastoma to DNA-Damaging Treatments by Suppressing PI3K-Mediated Homologous Recombination Repair

Xin Peng, Shaolu Zhang, Yingying Wang, Zhicheng Zhou, Zixiang Yu, Zhenxing Zhong, Liang Zhang, Zhe-Sheng Chen, Francois X. Claret, Moshe Elkabets, Feng Wang, Fan Sun, Ran Wang, Han Liang*, Hou-Wen Lin* and Dexin Kong**

List of Items in Supporting Information

**Figure S1**	**STELB inhibits GBM cell proliferation and induces DNA damage**
**Figure S2**	**STELB sensitizes MGMT-negative GBM cells to TMZ**
**Figure S3**	**Effects of STELB on HR and NHEJ repair in GBM cells**
**Figure S4**	**Effects of STELB on PI3K activity and PI3K protein expression in GBM cells**
**Figure S5**	**Effects of genetic and pharmacological inhibition of PI3K on the expression of HR repair key factors BRCA1/2 and RAD51**
**Figure S6**	**Effects of STELB on mRNA expression of PI3K isoforms and the stability of exogenous PI3Kα**
**Figure S7**	**Effects of genetic and pharmacological inhibition of PI3K on HR repair efficiency**
**Figure S8**	**Effects of STELB on NHEJ-related proteins in vivo**
**Table S1**	**The primer sequences used in this study**

Supplementary Figure 1

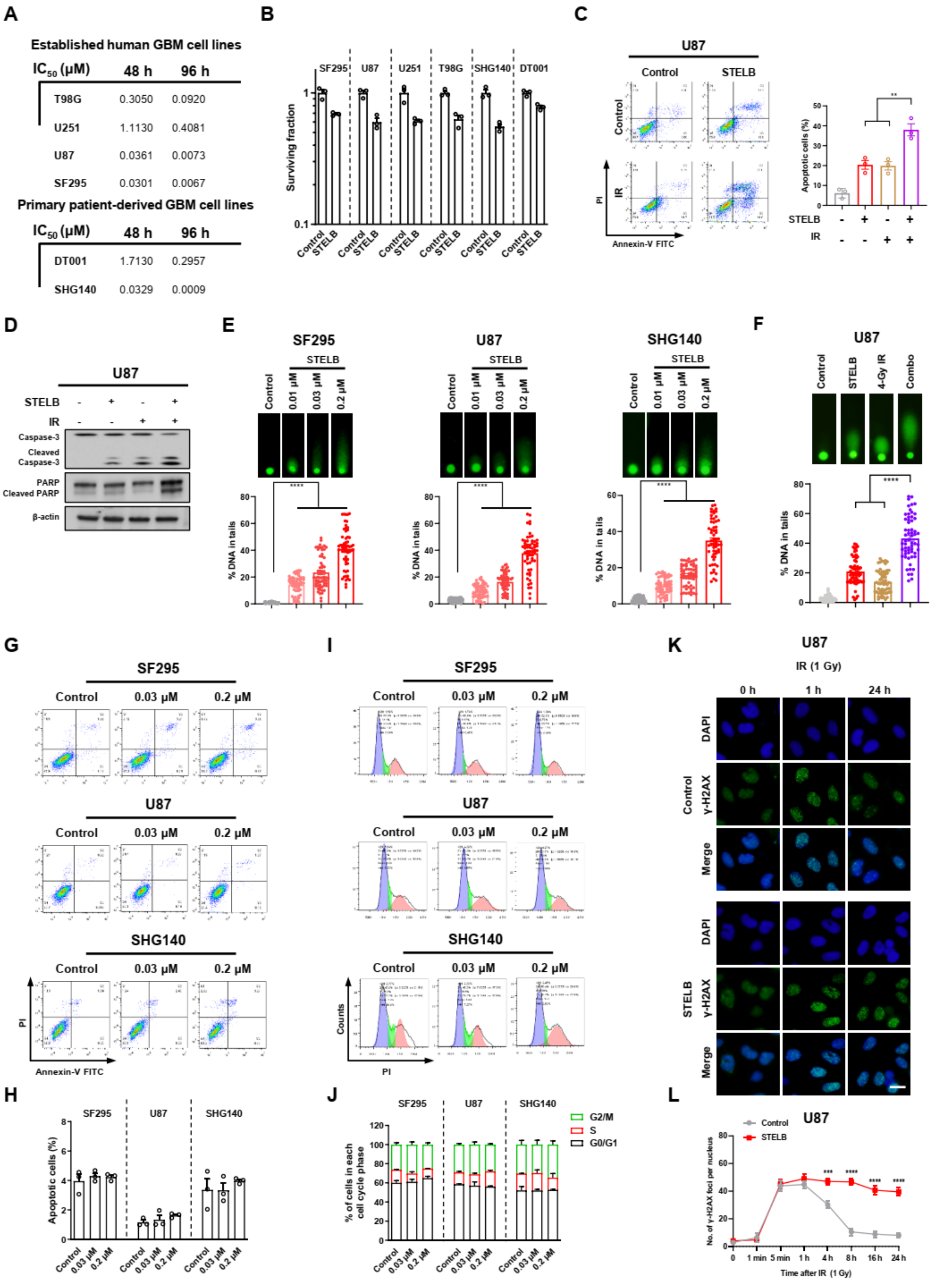

Figure S1. STELB inhibits GBM cell proliferation and induces DNA damage

(**A**) IC_{50} for established GBM cell lines and primary patient-derived GBM cell lines treated with increasing concentrations of STELB for 48 h or 96 h. (**B**) Clonogenic survival assay for GBM cells with or without pretreatment of STELB. (**C**) Apoptosis assays of U87 cells pretreated with STELB for 24 h and then irradiated at 4 Gy. Apoptosis was detected by Annexin V/PI staining after 72 h. (**D**) Western blot analysis of Caspase-3 and PARP in U87 cells after treatment with STELB and/or IR for 48 h. (**E**) Alkaline comet assays of U87 cells treated with different concentrations of STELB for 48 h to measure both single and double stranded DNA breaks. The selected doses are the IC_{50} value of STELB in the three cell lines for 48 h, a dose below the IC_{50} value, and a higher dose above the IC_{50} value. (**F**) Alkaline comet assays of U87 cells treated with STELB and/or IR for 48 h to measure both single and double stranded DNA breaks. The % DNA in tails was quantified to indicate the degree of DNA damage. (**G**) SF295, U87 and SHG140 cells treated with STELB for 24 h and then apoptosis was detected by Annexin V/PI staining. (**H**) FACS quantification of the total apoptotic cell population, including Annexin V^{+}/PI^{-} early apoptotic cells and Annexin V^{+}/PI^{+} late apoptotic cells. (**I**) Cell cycle analysis of SF295, U87 and SHG140 cells treated with different concentrations of STELB for 48 h. (**J**) The percentage of cell population at G0/G1, S and G2/M phases. (**K**) γ-H2AX foci assays of U87 cells treated with STELB and/or a 1 Gy dose of IR. The formation and resolution of γ-H2AX foci were assessed using immunofluorescence. Scale bar, 20 µm. (**L**) Quantification of the number of γ-H2AX foci per nucleus at each time point. Representative images are shown. Graphs are shown as the mean ± SEM from three independent experiments; P-values, two-tailed unpaired Student's t-test; *p < 0.05; **p < 0.01; ***p < 0.001; **** p < 0.0001.

Supplementary Figure 2

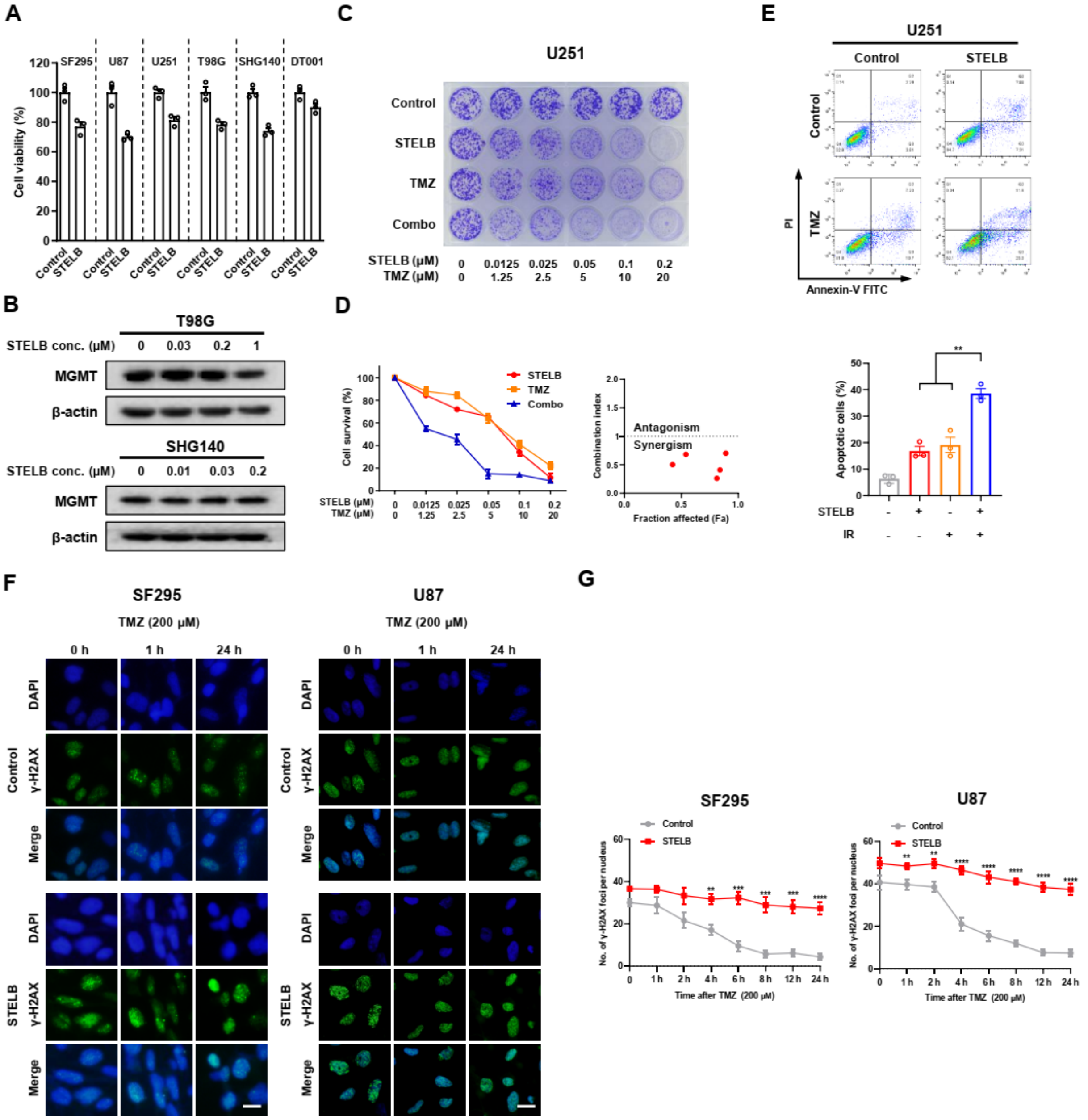

Figure S2. STELB sensitizes MGMT-negative GBM cells to TMZ

(**A**) Cell viability assay for GBM cells with or without pretreatment of STELB. (**B**) Western blot analysis of MGMT in T98G and SHG140 cells after treatment with STELB for 48 h. (**C**) Long-term clonogenic assays for U251 cells treated with STELB and/or TMZ for 72 h, allowed to recover for 10-15 days, and then subjected to crystal violet staining. Colony formation was observed under a microscope. (**D**) Quantification of (**C**). The absorbance at 570 nm was measured after incubation with 1% SDS for 3 h. Cell survival (%) is expressed as % of the control; CIs were calculated using CalcuSyn. CI < 0.9 represents synergism, 0.9 < CI < 1.1 represents additivity, and CI > 1.1 represents antagonism. (**E**) Apoptosis assays for U251 cells treated with STELB and/or 100 μM TMZ for 72 h. Apoptosis was detected by Annexin V/PI staining. FACS quantification of the total apoptotic cell population, including Annexin V^+/PI^- early apoptotic cells and Annexin V^+/PI^+ late apoptotic cells. (**F**) γ-H2AX foci assays of SF295 and U87 cells treated with STELB and/or 200 μM TMZ for 24h, then the STELB/TMZ contained culture media were replaced with common culture media. The formation and resolution of γ-H2AX foci were assessed using immunofluorescence. Scale bar, 20 μm. (**G**) Quantification of the number of γ-H2AX foci per nucleus at each time point. Representative images are shown. Graphs are shown as the mean ± SEM from three independent experiments; P-values, two-tailed unpaired Student's t-test; ***p < 0.001; ****p < 0.0001.

Supplementary Figure 3

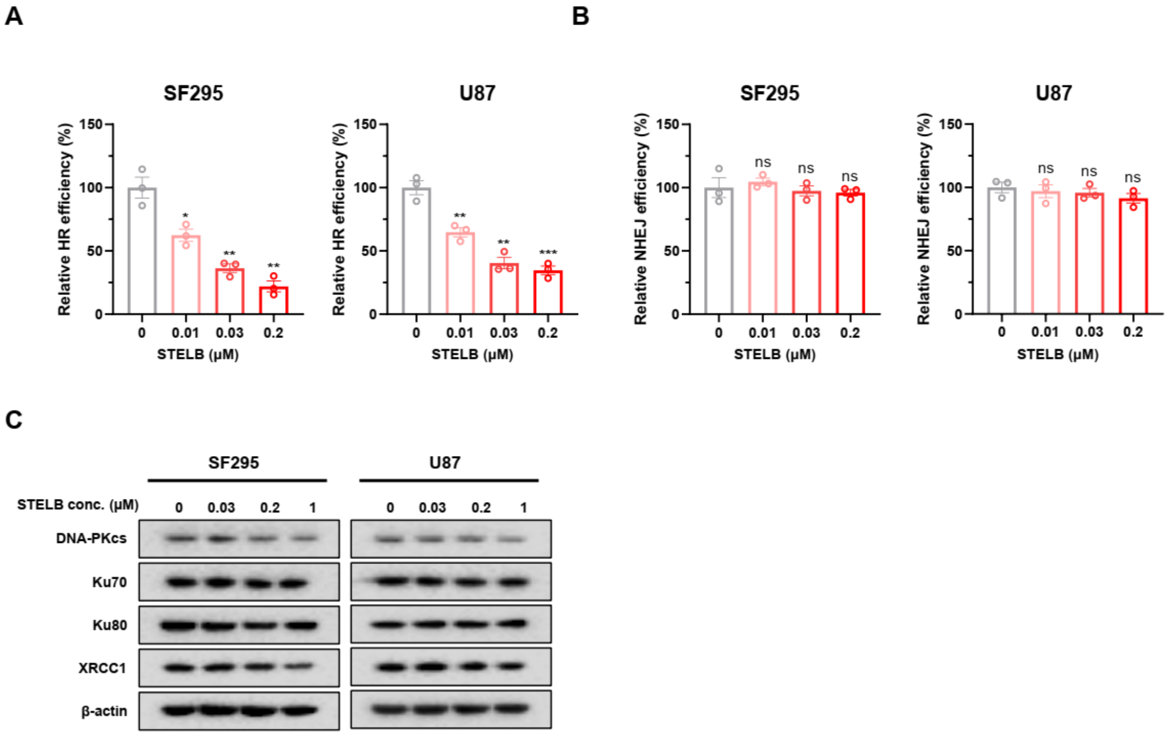

Figure S3. Effects of STELB on HR and NHEJ repair in GBM cells

(**A**) HR reporter assays to evaluate the effect of STELB on HR repair efficiency in SF295 and U87 cells. (**B**) NHEJ reporter assays to evaluate the effect of STELB on NHEJ repair efficiency in SF295 and U87 cells. (**C**) Western blot analysis of key proteins involved in the NHEJ repair pathway in SF295 and U87 cells treated with STELB for 48 h. Graphs are shown as the mean ± SEM from three independent experiments; P-values, two-tailed unpaired Student's t-test; *$p < 0.05$; **$p < 0.01$; ***$p < 0.001$.

Supplementary Figure 4

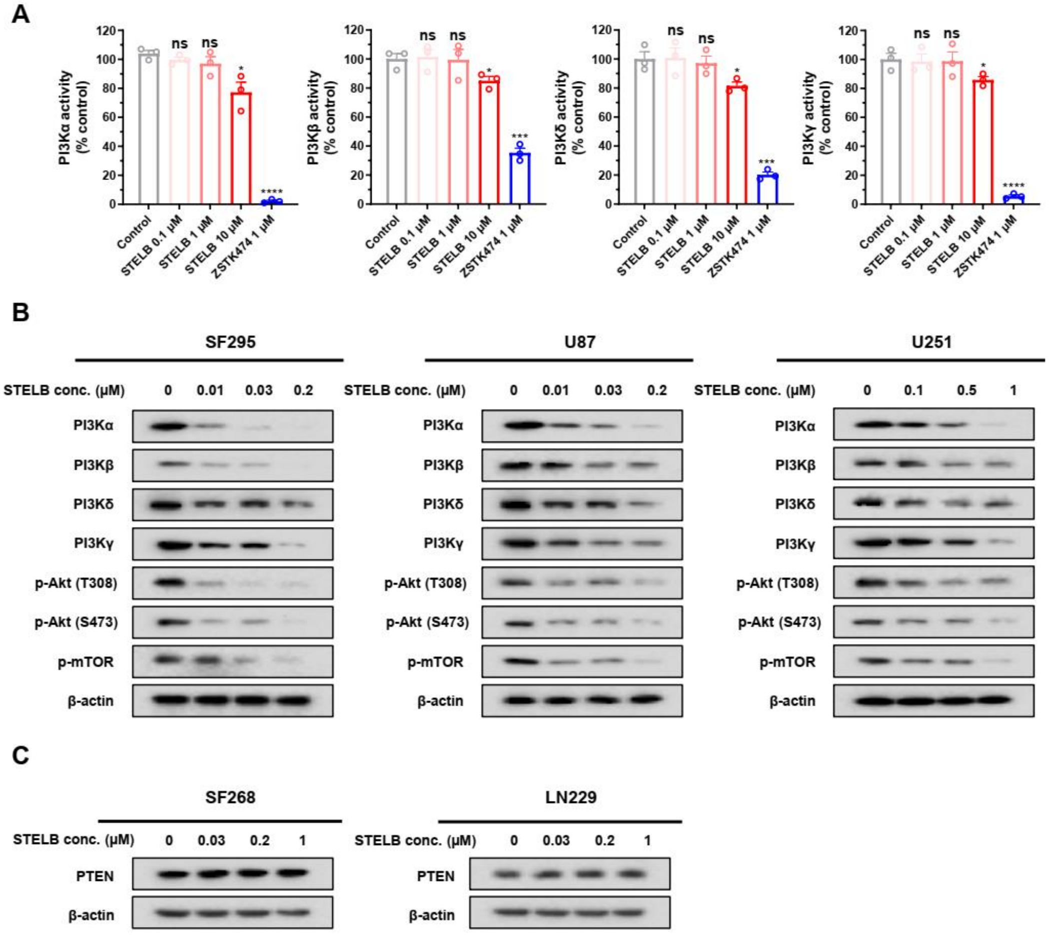

Figure S4. Effects of STELB on PI3K activity and PI3K protein expression in GBM cells

(**A**) Kinase activities of the four PI3K isoforms after co-incubation with the indicated concentrations of STELB. Pan-PI3K inhibitor ZSTK474 serves as a positive control. (**B**) Western blot analysis of the effects of STELB treatment on proteins involved in the PI3K pathway in GBM cells. (**C**) Western blot analysis of PTEN in the PTEN-WT GBM SF268 and LN229 cell lines treated with STELB for 48 h. Graphs are shown as the mean ± SEM from three independent experiments; P-values, two-tailed unpaired Student's t-test; ns indicates not significant; *$p < 0.05$; ****$p < 0.0001$.

Supplementary Figure 5

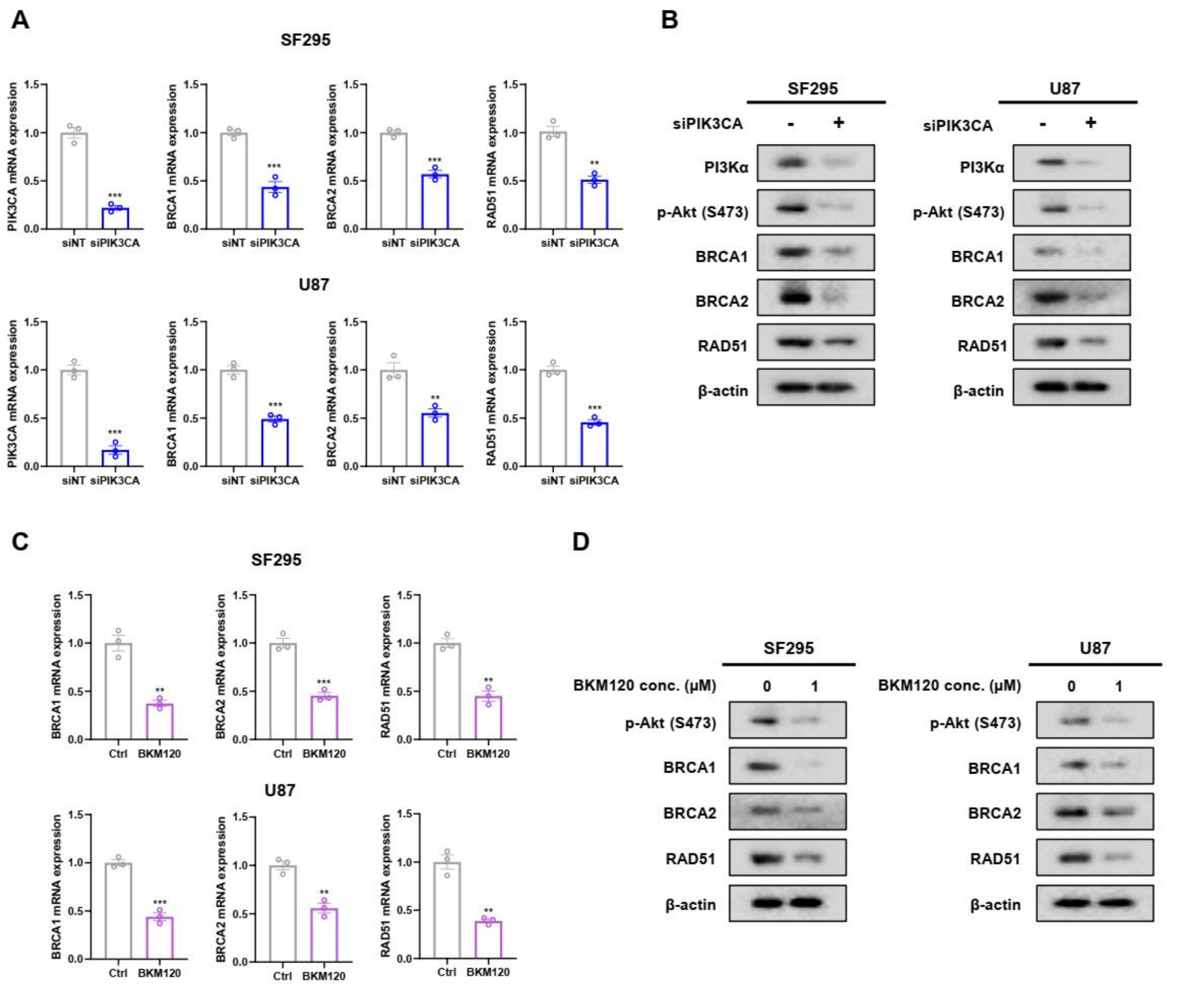

Figure S5. Effects of genetic and pharmacological inhibition of PI3K on the expression of HR repair key factors BRCA1/2 and RAD51

qRT-PCR analysis (**A**) and Western blot analysis (**B**) of SF295 and U87 cells transfected with siPIK3CA or siNT to quantify the HR repair key factors BRCA1/2 and RAD51. qRT-PCR analysis (**C**) and Western blot analysis (**D**) of SF295 and U87 cells treated with PI3Ki BKM120 to quantify BRCA1/2 and RAD51. Graphs are shown as the mean ± SEM from three independent experiments; P-values, two-tailed unpaired Student's t-test; **p < 0.01; ***p < 0.001.

Supplementary Figure 6

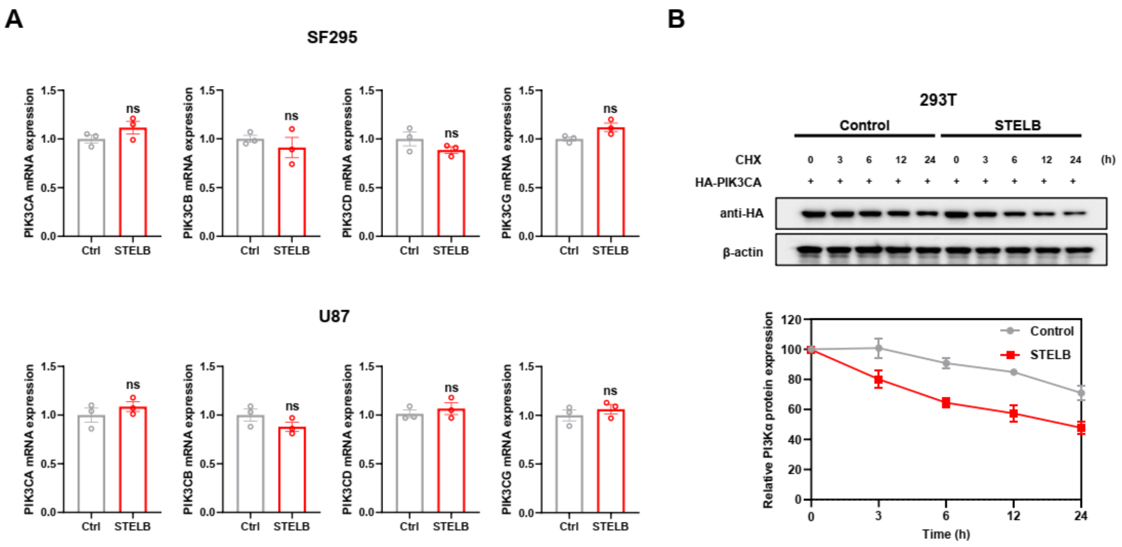

Figure S6. Effects of STELB on mRNA expression of PI3K isoforms and the stability of exogenous PI3Kα

(**A**) qRT-PCR analysis of SF295 and U87 cells treated with STELB to quantify mRNA levels of four PI3K isoforms, PIK3CA, PIK3CB, PIK3CG, and PIK3CD. (**B**) Stability analysis of exogenous PI3Kα protein in 293T cells transfected with HA-PIK3CA. Cells were treated with 20 μM CHX for indicated durations and analyzed by Western blot. Graphs are shown as the mean ± SEM from three independent experiments; P-values, two-tailed unpaired Student's t-test; ns indicates not significant.

Supplementary Figure 7

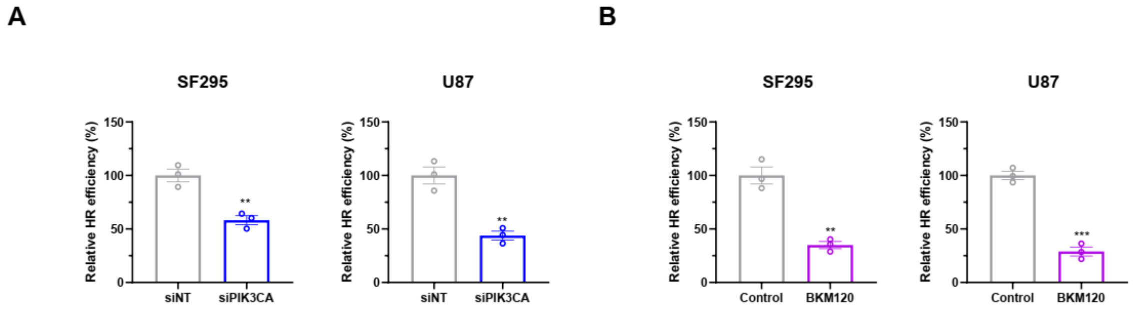

Figure S7. Effects of genetic and pharmacological inhibition of PI3K on HR repair efficiency

HR reporter assays to detect the effect of siRNA knockdown of PIK3CA (**A**) or BKM120 treatment (**B**) on HR repair efficiency in SF295 and U87 cells. Graphs are shown as the mean ± SEM from three independent experiments; P-values, two-tailed unpaired Student's t-test; $**p < 0.01$; $***p < 0.001$.

Supplementary Figure 8

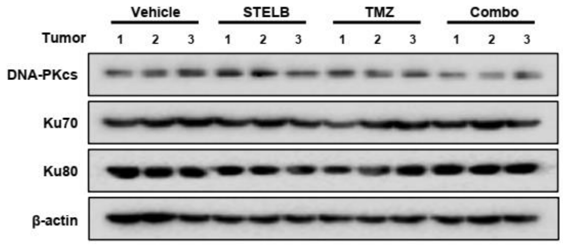

Figure S8. STELB did not influence NHEJ related proteins in vivo.

Western blot analysis of DNA-PKcs, Ku70, and Ku80 in tumor lysates. Three representative samples were selected from each group.

Table S1. The primer sequences used in this study.

Genes	Sequence (5′-3′)
PIK3CA	Forward: CGGTGACTGTGTGGGACTTATTGAG<br>Reverse: TGTAGTGTGTGGCTGTTGAACTGC
PIK3CB	Forward: GAGATTGCAAGCAGTGATAGTG<br>Reverse: TAATTTTGGCAGTGATTGTGGG
PIK3CD	Forward: GACCCAGAAGTGAACGACTTTC<br>Reverse: CCTCAAACTTAACGTTGACCAG
PIK3CG	Forward: CACCCAAAAGCATATCCTAAGC<br>Reverse: GTAATGCAGAACATCATCGTCC
BRCA1	Forward: GAACGGGCTTGGAAGAAAAT<br>Reverse: GTTTCACTCTCACACCCAGA
BRCA2	Forward: CAGGTAGACAGCAGCAAGCA<br>Reverse: AAGCCCCTAAACCCCACTTC
RAD51	Forward: CAGATGCAGCTTGAAGCAAA<br>Reverse: TTCTTCACATCGTTGGCATT
MRE11	Forward: TCAGATCTCAGTCAGAGGAGTC<br>Reverse: AGCCATCTGTTCTGCTAAATCT
RAD54B	Forward: TTATCCACGAACTTCGACCTAC<br>Reverse: CCACCAGCTTTTGAACTTAACA
BRIP1	Forward: GTGTGCCAGACTGTGAGCCAAG<br>Reverse: GCCATAAACCAGTAGAGAGCCAACG
FANCD2	Forward: GCATCTTCCTCCACATTCCCTACAC<br>Reverse: CACCGTCTTCTCTAGTTCAGCCATC
FANCM	Forward: AAACGTCAAGGCAGGATAGTTA<br>Reverse: GATAGATGACTTTCGCTGCAAG
18S rRNA	Forward: CAGCCACCCGAGATTGAGCA<br>Reverse: TAGTAGCGACGGGCGGTGTG
